# Long-term effect of sodium selenite on the integrity and permeability of on-chip microvasculature

**DOI:** 10.1063/5.0122804

**Published:** 2022-11-14

**Authors:** Maneesha Shaji, Atsuya Kitada, Kazuya Fujimoto, Stanislav L. Karsten, Ryuji Yokokawa

**Affiliations:** Department of Micro Engineering, Kyoto University, Kyoto Daigaku-Katsura, Nishikyo-ku, Kyoto 615-8540, Japan

## Abstract

Development of the robust and functionally stable three-dimensional (3D) microvasculature remains challenging. One often-overlooked factor is the presence of potential anti-angiogenic agents in culture media. Sodium selenite, an antioxidant commonly used in serum-free media, demonstrates strong anti-angiogenic properties and has been proposed as an anticancer drug. However, its long-term effects on *in vitro* microvascular systems at the concentrations used in culture media have not been studied. In this study, we used a five-channel microfluidic device to investigate the concentration and temporal effects of sodium selenite on the morphology and functionality of on-chip preformed microvasculature. We found that high concentrations (∼3.0 *μ*M) had adverse effects on microvasculature perfusion, permeability, and overall integrity within the first few days. Moreover, even at low concentrations (∼3.0 nM), a long-term culture effect was observed, resulting in an increase in vascular permeability without any noticeable changes in morphology. A further analysis suggested that vessel leakage may be due to vascular endothelial growth factor dysregulation, disruption of intracellular junctions, or both. This study provides important insight into the adverse effects caused by the routinely present sodium selenite on 3D microvasculature in long-term studies for its application in disease modeling and drug screening.

## I. INTRODUCTION

Vasculature is an essential component for the proper function and development of different organs.^1^ It is critical for homeostasis, oxygen and nutrient exchange, and the removal of waste products for maintaining viable tissues. Vasculature also plays an important role in various disease pathophysiology^2,3^ and treatment.^1,4^ Therefore, *in vitro* recapitulation of physiologically relevant three-dimensional (3D) vascular systems is of great importance in clinical studies.

With advances in microfluidic technology, complex 3D microvasculature can now be constructed.^5^ While microvasculature can be formed either by pre-patterning or self-assembly of endothelial cells in these platforms, vasculature formed by the self-assembly method closely resembles the process of *in vivo* vascularization. In this method, the functional and perfusable vascular structures are formed by vasculogenesis and/or angiogenesis.^6^ Irrelevant of what approach is used, critical parameters of *in vitro* formed microvasculature include its consistent perfusability and long-term integrity preventing undesirable leakage.

Vasculature on-chip provides tremendous flexibility in the customization of the microenvironment through accurate control of biological, mechanical, biochemical, and biophysical parameters.^5–7^ Different cell types can be easily integrated for direct and indirect interactions in microfluidic compartments, along with fluid flow conditions. Organ-specific vasculatures can also be studied. For example, studies have shown the construction of a blood–brain barrier consisting of endothelial cells, pericytes, and astrocytes in a microfluidic system, for the elucidation and evaluation of complex transport mechanisms of solute and molecules across the barrier.^8^ Additionally, stem cells can be used for the creation of *in vivo*-like vascular networks to precisely represent human biology. Such *in vitro* platforms have also been shown to simulate vascular defects in many disease conditions such as hemorrhagic telangiectasia,^9^ brain arteriovenous malformations,^10^ tumor,^11^ and SARS-CoV-2-induced inflammation.^12^ Furthermore, vasculature on-chip can be integrated with organoids for the creation of vascularized organoid models.^7^ For example, vascularized tumor on-chip models have been demonstrated for drug delivery applications.^13^ Thus, these platforms are excellent tools to study complex vascular physiology with the real-time observation of different biological processes, including cellular interactions and barrier functions for developmental studies as well as drug delivery applications.^5–7^

Construction of a realistic and physiologically relevant vasculature on-chip requires careful selection and tuning of the endothelial cells, supporting cells, scaffolds, tissue-specific extracellular matrix, and culture media along with biochemical and biophysical cues.^5,6^ Although many studies have been reported for the optimization of the above-mentioned factors, the presence of anti-angiogenic components in the culture media is surprisingly often neglected and understudied. In this study, we investigated the effects of one such commonly used supplement, Sodium Selenite (SS). In mammalian cell cultures, including stem cells, selenium, an essential trace element, is used in the form of SS (from 30 to 40 nM) because of its antioxidative properties.^14^ It reduces the production of reactive oxygen species (ROS) and lipid peroxidation, which normally occurs in high-oxygen culture conditions.^14,15^ However, SS also possesses cytotoxic, anti-proliferative, and anti-angiogenic properties, making it a potential drug candidate for cancer treatment.^16^ SS exerts its anti-cancer effect by inhibiting angiogenesis^17–20^ and/or inducing apoptosis^21–23^ in prostate,^18,24^ mammary,^19–22^ and liver^17,25^ cancers. SS is known to oxidize cell membrane thiols, preventing the formation of insoluble, protease-resistant fibrin-like polymer coats around solid tumors. As a result, cancer cells become vulnerable to immune surveillance and destruction.^16,26^ The anti-angiogenic behavior of SS is regulated by suppressing vascular endothelial growth factor (VEGF) and matrix metalloproteinase 2 (MMP-2), two crucial proteins required for angiogenesis.^27^ By contrast, selenite treatment has been shown to induce angiogenesis and increase VEGF levels to reduce endothelial dysfunction in diabetes.^28^ However, the action mechanisms of selenite and SS on blood vessels remain unclear.

The main objective of this study was to evaluate the temporal and concentration-dependent effects of SS on the morphology and integrity of microvascular networks-on-chip. A five-channel microfluidic system was used to create microvasculature formed by the self-organization of green fluorescent protein (GFP)-expressing human umbilical vein endothelial cells (HUVECs). Furthermore, gene expression of different angiogenic and adhesion factors was investigated to identify the molecular mechanisms involved.

## II. RESULTS

### A. High concentration of sodium selenite degrades microvasculature within 6 days

**FIG. 1.**
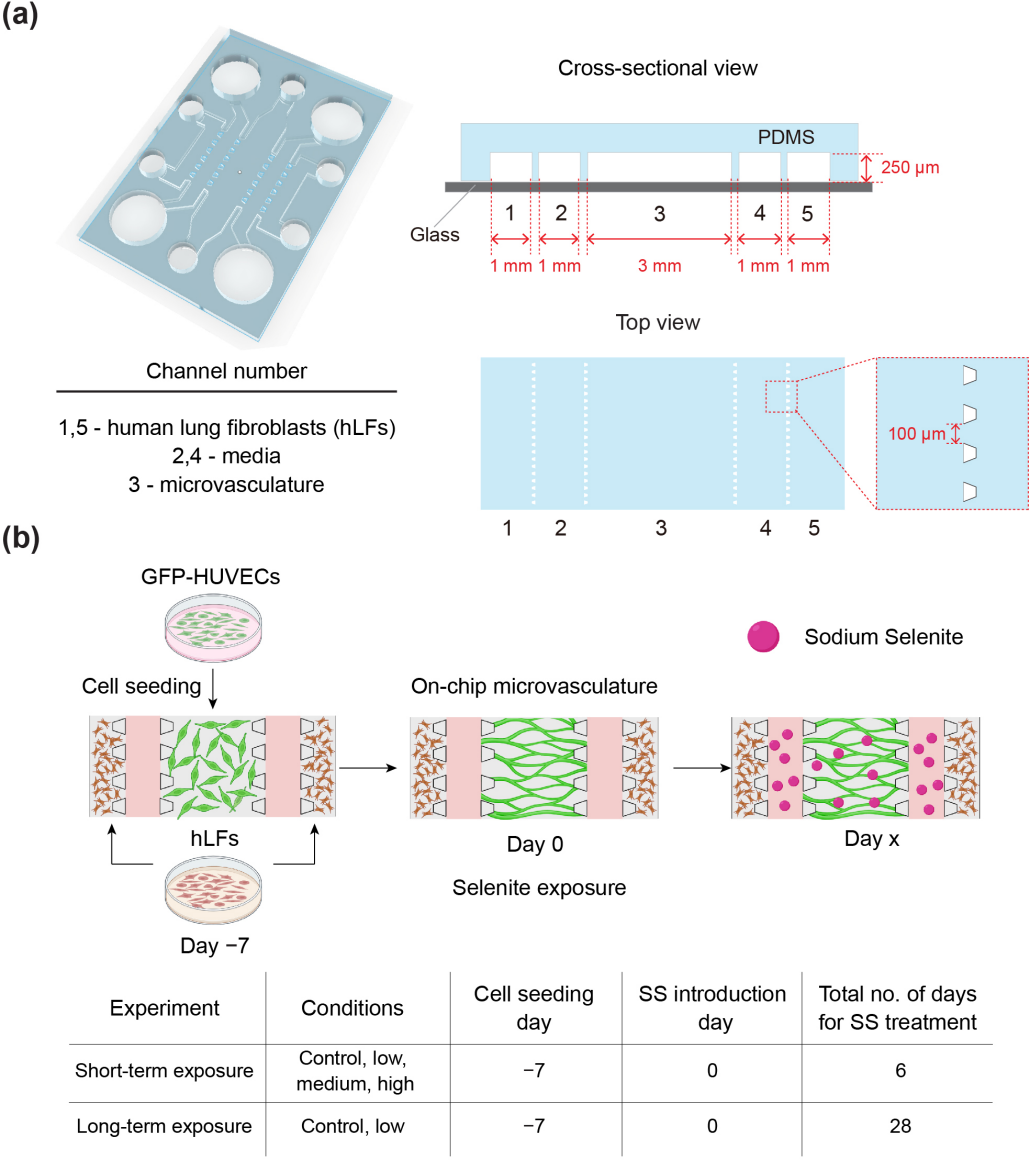
Experimental setup to study the effect of sodium selenite (SS) on on-chip microvasculature. (a) Five-channel microfluidic device, consisting of a top PDMS layer and a bottom glass layer. (b) Schematics and table showing the experimental procedure to study the effects of SS on on-chip microvasculature. The day on which SS was introduced on chip was denoted as day 0. SS was administrated for up to 6 days and 28 days (day x) for short-term and long-term exposure experiments, respectively.

To create the on-chip microvasculature, a five-channel microfluidic device was employed [Fig. 1(a)]. Perfusable and functional microvascular networks were formed on-chip within 7 days by the self-organization of HUVECs in the presence of hLFs [Fig. 1(b)]. The on-chip microvasculature was then exposed to different SS concentrations (0, 3, 15 nM, and 3 *μ*M) for up to 28 days [Fig. 1(b)].

**FIG. 2.**
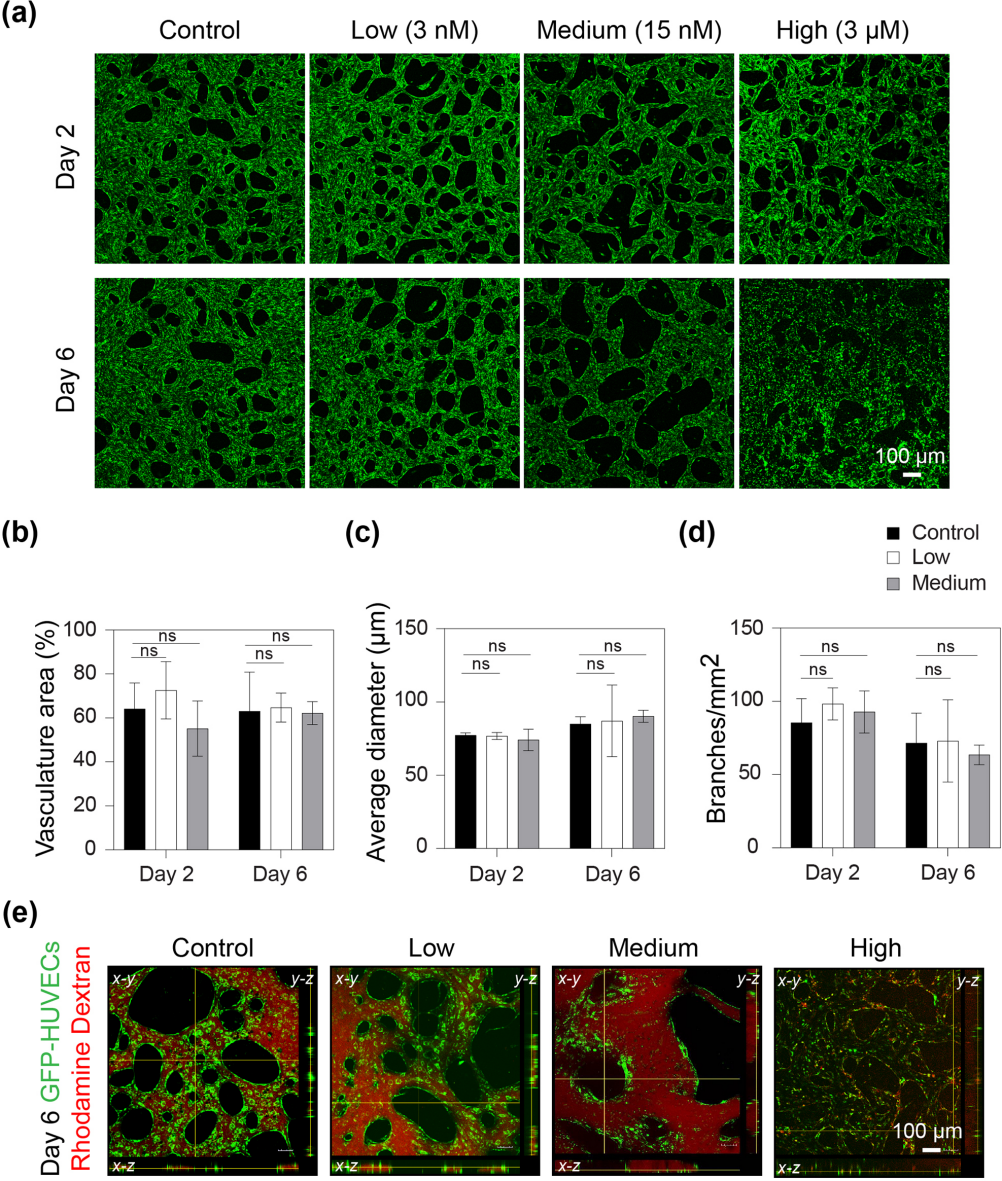
Short-term exposure of on-chip microvasculature to sodium selenite (SS). (a) Fluorescent images showing the on-chip microvasculature (shown in green) morphology, for control (0 nM), low (3 nM), medium (15 nM), and high (3 *μ*M) SS concentrations at days 2 and 6 after exposure. (b) Percentage of area covered by microvasculature for control, low, and medium SS conditions at days 2 and 6, n = 3. (c) Average diameter of the microvasculature for control, low, and medium conditions at days 2 and 6, n = 3. (d) Branches per square millimeters of the microvasculature for control, low, and medium concentrations at days 2 and 6, n = 3. ns denotes no significance. High SS concentration (3 *μ*M) was not included in the quantitative analysis of vascular morphology in (b)–(d). (e) Confocal images showing the perfusability of the microvascular networks for control, low, medium, and high SS conditions, when fluorescent rhodamine dextran was introduced in one of the medium reservoirs, at day 6.

First, low (3 nM), medium (15 nM), and high (3 *μ*M) SS concentrations were tested for 6 days on chip (Fig. 2). Within 6 days, the microvasculature treated with high SS showed visible cell death [Fig. 2(a)], which resulted in the disruption of microvascular networks, with a partial to full loss of vascular integrity. This observation was validated by testing the perfusion of rhodamine dextran, a fluorescent dye, through the microvasculature. We defined perfusability as the ability of a preformed vascular network to allow the flow of dye from one side to the other. In the microvascular network exposed to a high SS concentration, the fluorescent dextran diffused uniformly through the gel and was not perfused through the vessel-like walls, pointing to a physical degradation of the existing microvasculature [Fig. 2(e)]. The cytotoxicity of high SS concentrations was also confirmed with regular dish cultures, which showed a significant increase in cell death within 24 h of SS treatment (Fig. S1).

The exposure of the microvessels to low (3 nM) SS concentrations did not produce any visible morphological changes compared with the control (no SS). However, exposure to medium (15 nM) SS concentrations resulted in vessel hyperplasia compared with the control (no SS). No significant differences were observed in the microvasculature area [Fig. 2(b)], average microvascular diameter [Fig. 2(c)], and number of branches per square millimeter [Fig. 2(d)]. Moreover, the microvasculature exposed to low and medium SS concentrations remained perfusable even after 6 days of exposure [Fig. 2(e)]. Additionally, low and medium SS concentrations did not affect cell viability of endothelial cells cultured in regular culture dishes (Fig. S1). ROS production on-chip did not show any significant differences between the control and SS-treated samples (Fig. S2).

### B. Long-term exposure to low concentrations of sodium selenite increases microvascular permeability

**FIG. 3.**
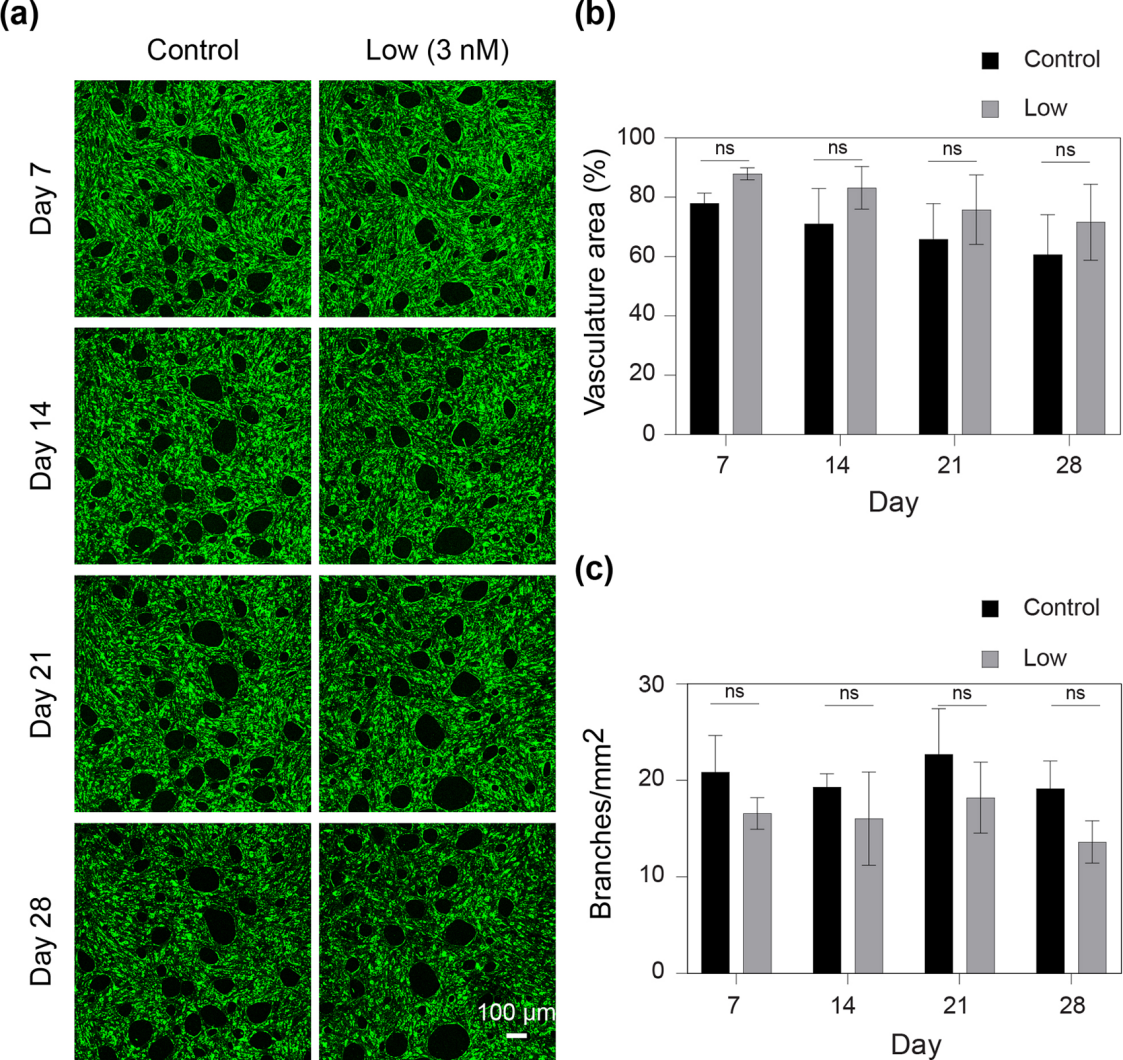
Long-term exposure of the preformed microvasculature to sodium selenite (SS) does not alter morphology. (a) Fluorescent images showing the on-chip microvasculature (green) morphology, for control (no SS) and low (3 nM) SS at 7, 14, 21, and 28 days of exposure. (b) Percentage of area covered by microvasculature for control and low SS at 7, 14, 21, and 28 days, n = 4. (c) Branches per square millimeters of the microvasculature for control and low SS after 7, 14, 21, and 28 days of SS exposure, n = 4.

The effects of very low SS concentrations (3 nM) were investigated for up to 28 days on chip (Fig. 3). First, no visible morphological changes were observed compared with the control [Fig. 3(a)], which was confirmed by a quantitative analysis [Figs. 3(b) and 3(c)]. Additionally, no significant changes were observed in the vasculature area [Fig. 3(b)] and the number of branches per square millimeter in the preformed microvasculature [Fig. 3(c)].

**FIG. 4.**
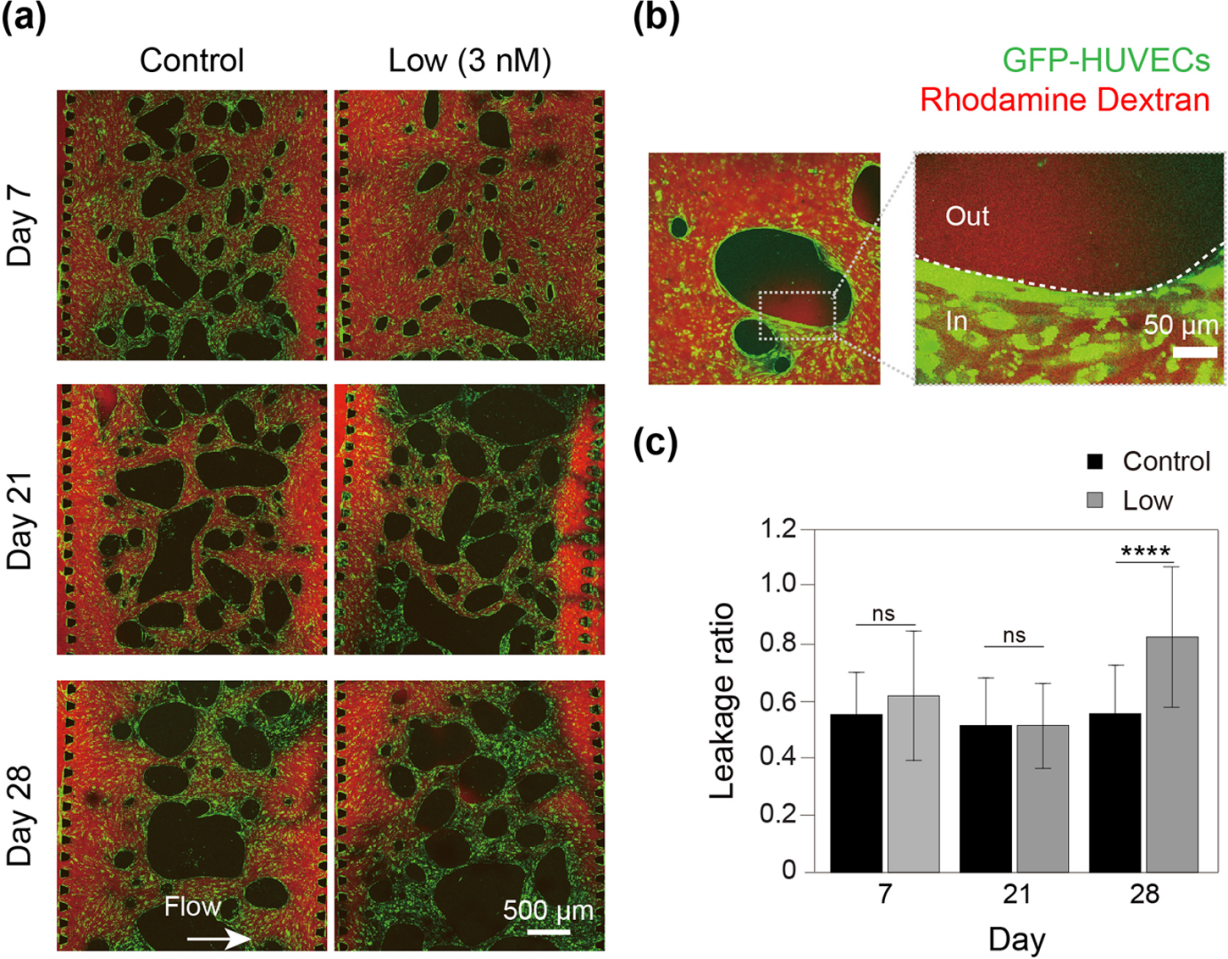
Long-term exposure of sodium selenite (SS) increases microvasculature permeability. (a) Fluorescent images showing the microvasculature (green) and fluorescent rhodamine dextran (red) for control (no selenite) and low SS (3 nM) after 7, 21, and 28 days of exposure. The white arrow shows the direction of dextran flow. (b) Enlarged representative images showing GFP positive vasculature area, with dextran inside (denoted as in) and outside the vessels (denoted as out). The white dotted line shows the vessel wall. (c) The leakage ratio of rhodamine dextran (out/in) in the on-chip microvasculature for control and low SS after 7, 21, and 28 days of treatment, n = 4. ^****^p < 0.0001 and ns—no significance.

The perfusability and permeability on days 7, 21, and 28 are shown in Fig. 4. The microvasculature remained perfusable even after 28 days of SS treatment [Fig. 4(a)]. The permeability of the microvasculature was examined through dye leakage from the inside of the vessel to the outside of the gel [Fig. 4(b)]. No significant changes in permeability or leakage were observed until day 21. However, on day 28 of SS exposure, permeability significantly increased [p < 0.0001; Fig. 4(c)].

### C. Sodium selenite exposure alters vascular permeability and cell adhesion properties

**FIG. 5.**
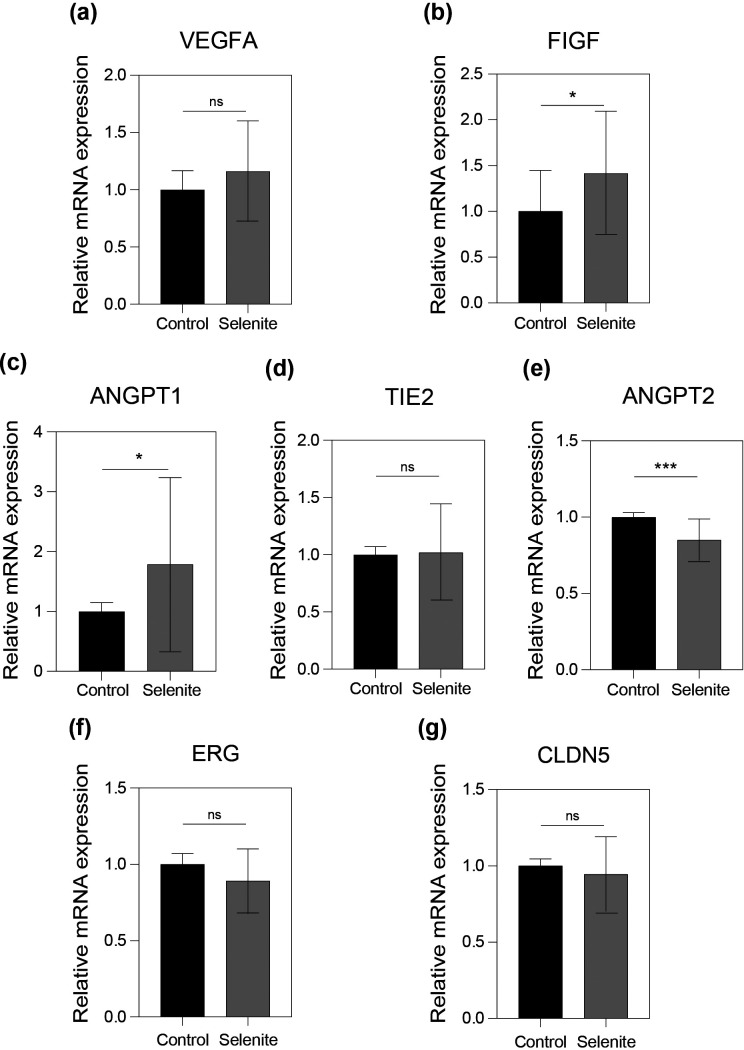
Relative gene expression analysis by quantitative RT-PCR. The mRNA expressions of (a) vascular endothelial growth factor A or VEGFA, (b) vascular endothelial growth factor D or FIGF, (c) angiopoietin 1 or ANGPT1, (d) TEK receptor tyrosine kinase or TIE2, (e) angiopoietin 2 or ANGPT2, (f) ETS-related gene or ERG, and (g) claudin 5 or CLDN5 of the on-chip microvasculature with sodium selenite (SS) (3 nM) relative to control (0 nM) at day 28 of SS treatment, n = 4. ^*^p < 0.05, ^***^p < 0.001, and ns—no significance.

**FIG. 6.**
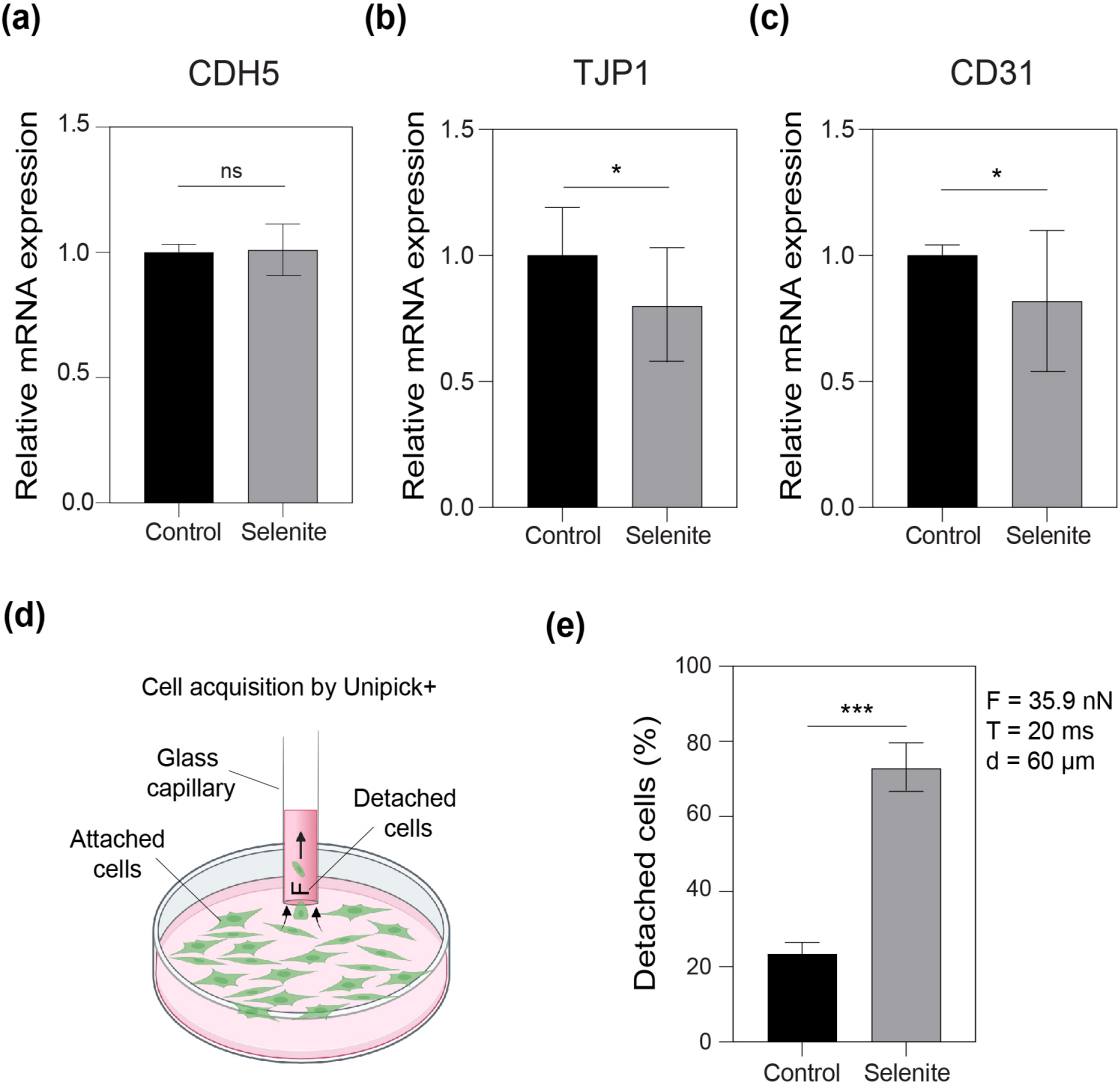
Sodium selenite (SS) alters cell adhesion properties. The mRNA expressions of (a) vascular endothelial cadherin or CDH5, (b) tight junction protein 1 or TJP1, and (c) platelet endothelial cell adhesion molecule or CD31 of the microvasculature with SS (3 nM) with respect to control (0 nM) at day 28 of SS treatment, n = 4. ^*^p < 0.05; ^***^p < 0.001; and ns denotes no significance. (d) Schematic diagram showing the cell acquisition by UnipicK+ to measure cell adhesion properties of GFP-HUVECs with and without SS. (e) Short term (4–7 h) exposure to high SS (3 *μ*M) significantly decreases cell–surface adhesion strength, n = 3. Cells were collected using a capillary with an internal diameter of 60 *μ*m, an acquisition pressure of −12 kPa, a force of 35 nN, and an acquisition pulse of 20 ms. ^***^p < 0.001.

To elucidate the mechanism by which SS alters vessel permeability, the expression of key genes related to vascular permeability or barrier function was examined on day 28 using quantitative reverse transcription-polymerase chain reaction (RT-PCR). The expression of seven genes was evaluated [Figs. 5 and 6(a)–6(c)].

Vascular endothelial growth factor A (VEGFA) expression showed no significant difference compared to control after SS treatment [Fig. 5(a)]. Whereas a significant increase in cellular-fos proto-oncogene (c-FOS)-induced growth factor (FIGF) expression [Fig. 5(b); fold change: 1.5] and angiopoietin 1 (ANGPT1) expression [Fig. 5(c); fold change >1.5] were observed at low SS concentrations after 28 days. However, no significant regulation in gene expression was observed for tyrosine-protein kinase receptor (TIE2) and angiopoietin 2 (ANGPT2) compared with the control [Figs. 5(d) and 5(e)]. Furthermore, no significant changes were observed in the mRNA levels of the erythroblast transformation-specific (ETS)-related gene (ERG) [Fig. 5(f)], claudin 5 (CLDN5) [Fig. 5(g)], and cadherin 5 (CDH5) [Fig. 6(a)]. However, the SS-treated microvasculature showed a significant decrease in the expression of tight junction protein 1 (TJP1) [Fig. 6(b)] and platelet endothelial cell adhesion molecule (CD31) [Fig. 6(c)] in the preformed microvasculature on day 28.

To verify whether SS treatment can alter cell adhesion properties, a cell surface adhesion test was conducted. ECs treated with 3 *μ*M SS and the controls were collected using a capillary-based vacuum-assisted cell acquisition system (UnipicK+) [Fig. 6(d)]. The control ECs showed significantly stronger attachment to the surface of the culture dish [threefold, p < 0.001; Fig. 6(e)] than the SS ECs.

## III. DISCUSSION

Vasculature plays an important role in maintaining healthy tissues and is also involved in the paracrine signaling required for the proper development and function of different organs.^1^ Recent advances in microfluidic technology have enabled the construction of physiologically similar, fully functional vascular networks on a chip.^5^ Such a system can be tailored to meet organ-specific characteristics using customized biomaterials, growth factors, mechanical forces, and culture media.^5,6^ In addition to optimizing these factors to promote cell/tissue growth, it is also vital to exclude components that may inhibit certain biological processes, such as angiogenesis.

SS is an angiogenesis inhibitor that has been used to treat cancer.^27,29^ It is also commonly used as an antioxidant in serum-free media for mammalian and stem cell cultures.^14^ However, the precise effects of selenite on *in vitro* vasculature are unknown. In this study, we utilized a simple yet efficient method to investigate the effects of SS on a preformed 3D vasculature using a microfluidic device (Fig. 1). This method ensured the singular evaluation of the effect of SS on *in vitro* microvasculature.

Our findings confirmed the cytotoxic effect of SS at micromolar concentrations (Figs. 2 and S1). This result is in agreement with previous reports, which demonstrated that high concentrations of SS are toxic and may even be used to treat cancer.^15,16,18^ Serum-free media typically contains nanomolar concentrations of SS.^30,31^ Although these concentrations are markedly lower than the reported toxic SS concentrations (>5 *μ*M),^15,16,21,22^ it is important to evaluate their effects on *in vitro* microvasculature. In this study, we report for the first time that long-term exposure to even seemingly advantageous nanomolar SS concentrations could increase vascular permeability, leading to abnormal vessel characteristics (Figs. 4–6).

Since a high SS concentration was toxic, this condition was omitted from the morphological analysis to avoid methodological artifacts due to remaining cell debris. Because selenite was originally used to reduce ROS production,^30^ ROS production on the chip was also evaluated; however, no significant differences were observed between the control and SS-treated samples (Fig. S2). Thus, short-term exposure to SS exerted toxic effects on the vasculature at high SS concentrations but demonstrated no adverse outcomes at low SS and medium SS concentrations (Fig. 2).

One of the major applications of *in vitro* vascular systems lies in organ-on-chip studies for developmental studies, drug discovery, and testing.^7^ To ensure that such an organ-on-chip system closely resembles its *in vivo* counterpart, long-term culturing spanning weeks to months is often a prerequisite.^7^ Hence, the evaluation of the long-term effects of SS on the microvasculature is of utmost importance (Figs. 3 and 4). We found that low SS concentrations resulted in increased permeability without any noticeable morphological changes when administered for 28 days. This is a very important result, because the vessels with seemingly normal morphology might be functionally deficient (leaky) in the long-term cultures.

The ability of the blood vessel wall to selectively allow the transport of ions and molecules across is essential for nutrient exchange, homeostasis, and protection of organs from foreign particles.^32^ Although permeability serves many physiological functions during wound healing,^33^ glomerular filtration,^34^ and embryonic development,^35^ dysfunction in permeability can also indicate the development of certain disease pathologies.^36^ For instance, in cancer or edema,^36^ blood vessels demonstrate high permeability owing to the presence of leaky vessels. It has also been shown that during diabetic macular edema,^37^ endothelial cells show fenestrations, which, in turn, leads to an increase in vascular permeability. Hence, vascular permeability is often considered a pathophysiological indicator of disease progression.

Since the primary goal of vascularized *in vitro* organ-on-chip models is to establish functional and perfusable vessel-like networks inside the tissue to facilitate maturation and removal of toxic substances; the increase in vessel permeability or leakage is a matter of concern, as it may lead to tissue dysfunction and death. In this respect, the stability and integrity of *in vitro* vessel-like networks are often overlooked in on-chip vascularization studies. Hence, our findings regarding the role of SS in the long-term increase in vessel permeability are of the greatest importance (Fig. 4).

We also examined changes in gene expressions related to vascular permeability at day 28 by quantitative RT-PCR, to understand the mechanism of SS action on the on-chip microvasculature. VEGFA [Fig. 5(a)], also known as the vascular permeability factor, is a well-known vasodilator and permeabilizing agent.^38^ VEGFA-mediated vascular permeability occurs either by the activation of VEGF receptors, VEGFR1, and VEGFR2, by increased calcium signaling, or by ultrastructural changes.^38^ Ultrastructural changes include cell junctional changes (usually loss of junctional integrity),^38^ formation of fenestrations,^39^ and formation of vesicular vacuole organelles,^40^ resulting in a long-term change in endothelial junctional properties. VEGFD or FIGF is another important VEGF protein. VEGFD binds to VEGFR2 and VEGFR3 and is known to increase vascular leakage or edema during lung injury.^41^ The significant increase in FIGF gene expression [Fig. 5(b)] might suggest FIGF-dependent vascular leakage in the presence of SS *in vitro*. However, the exact mechanism requires further investigation.

Furthermore, the expression of angiogenic factors related to vascular permeability was examined [Figs. 5(c)–5(e)]. The ANGPT-TIE pathway is a well-known pathway for vascular quiescence. In cardiovascular diseases, blood vessel leakage is induced by the inhibition of TIE2, mediated by ANGPT2. Additionally, ANGPT1 is known to control vascular stability by interacting with TIE receptors.^42^ The significant increase in ANGPT1 [Fig. 5(c)] could be an implication of a possible attempt by the microvasculature to regain its vascular stability and integrity during increased permeability conditions.

Vascular inflammation is another factor that can trigger endothelial barrier dysfunction. To examine whether SS induced inflammatory responses in the microvasculature, the expression of ERG and its downstream target CLDN5 was measured. ERG, which is highly enriched in ECs, and CLDN5 have been identified to play an important role in inflammation-induced vascular dysfunction, leading to increased permeability.^43^ The absence of any significant changes in both ERG and CLDN5 mRNA levels suggests the absence of an inflammatory response during SS treatment in the preformed vascular networks [Figs. 5(f) and 5(g)].

Vascular permeability is closely associated with the barrier function of the endothelial cell junctions. The strength and organization of the junctions control the blood vessel leakage. Junctions can be of different types: adherens and tight gap junctions. In ECs, the adherens and tight junctions are intermixed.^44^ Vascular endothelium-specific cadherin or CDH5 is one of the molecular components of the adherens junction and is expressed by all types of ECs, irrespective of the vessel type. Phosphorylation of VE-cadherin or CDH5 is also known to induce vessel leakage.^45^ However, no significant differences were observed in CDH5 expression [Fig. 6(a)].

Another possible mechanism resulting in the cleavage of EC junctions is the dissolution of VE-cadherin during apoptosis. The secretion of cadherin 1 or E-cadherin is one of the events that occur during this junction disruption.^46^ Since high concentrations of SS exhibit toxic behavior, we hypothesized that long-term exposure to very low concentrations might result in accumulated toxicity, which in turn might lead to apoptosis. Hence, to check for the dissolution of VE-cadherin, the gene expression of E-cadherin was examined. However, no expression was detected, eliminating the possibility of vascular leakage induced by the dissolution of VE-cadherin (data not shown).

Cell-to-cell adhesion depends on the localization of tight junction proteins (TJPs). TJPs are involved in the regulation of permeability through actin–myosin interactions.^47^ An increase in vessel permeability may also be achieved by phosphorylation of TJP1.^48^ It is tempting to speculate that the significant decrease in zonula occludens (ZO1) or TJP1 expression might be due to a possible ultrastructural change, such as the formation of tight junction pores in EC junction complexes [Fig. 6(b)]. Another cell adhesion molecule, CD31, which also shows a significant decrease in gene expression, is known to alter vessel permeability through its association with integrins.^44^ It has also been reported that the inactivation of CD31 results in increased permeability *in vivo.*^49^ Therefore, we hypothesized that the increased permeability of *in vitro* microvasculature exposed to low concentrations of SS in the long term might be due to alterations in cell adhesion properties with no visible morphological changes [Figs. 6(b) and 6(c)].

A similar phenomenon was pointed out in an *in vivo* study in which ECs with VE-cadherin −/− showed increased permeability without apparent morphological changes or organization of cell junctions.^50,51^ Therefore, a cell–surface adhesion test was conducted to verify whether SS treatment could alter cell adhesion properties [Fig. 6(d)]. The significant increase in detachment of ECs treated with 3 *μ*M SS compared with control ECs suggests that selenite exposure can indeed induce changes in cell adhesion properties, possibly affecting intracellular junctions and ultimately altering vessel wall permeability [Fig. 6(e)]. This is the first report that identified SS-induced vessel leakiness and changes in cell adhesion properties after long-term exposure to nanomolar quantities of SS. Hence, long-term exposure of the microvasculature to low concentrations of SS used in the standard cell culture conditions, resulted in increased vascular permeability, induced either by VEGFD, alterations in cell adhesion properties, or both, eventually transpiring to dysfunctional vessels.

## IV. CONCLUSIONS

Selenium, an essential component of serum-free media in the form of sodium selenite, also possesses anti-angiogenic properties, which may have adverse effects on preformed 3D microvasculature, leading to dysfunctional vessels. To our best knowledge, this is the first study to suggest that even significantly lower concentrations of SS in serum-free media can increase vascular permeability when administered for a long time. Initial experiments suggest that this might be caused either by VEGF-dependent leakage, prolonged alterations in cell adhesion, or both. The exact underlying molecular mechanisms remain unclear and require further studies. However, it is important to exercise caution and pay special attention to media content when designing microenvironments for *in vitro* use of preformed microvascular systems in drug delivery and other clinical studies.

## V. MATERIALS AND METHODS

### A. Microfluidic device

A polydimethylsiloxane (PDMS)-based two-layer, five-channel microfluidic device, fabricated using soft lithography, was used [Fig. 1(a)]. The channels consisted of five parallel microchannels separated by trapezoidal micropillars (width = 100 *μ*m and height = 250 *μ*m), which were fabricated by a photolithography process using SU8 2100 (MicroChem, USA) on a silicon wafer. The features were transferred to the PDMS (Silpot, Dow Corning Toray Co. Ltd.) top layer using a soft lithography process. Gel injection inlets (diameter = 2 mm) and medium reservoirs (diameter = 6 mm) were punched out from the PDMS layer using biopsy punches (sterile dermal biopsy punch; Kai Industries, Tokyo, Japan) of appropriate diameters. The PDMS top layer was then irreversibly bonded to a glass coverslip (Matsunami Glass, Osaka, Japan; 24 × 24 mm^2^) by plasma treatment (40 s, 50 W, flow rate of 50 sccm; Femto Science, Hwaseong, Korea) and baked at 120 °C overnight. The device was sterilized by UV treatment before the experiments.

### B. Cell and microvasculature preparation

Green fluorescent protein-expressing HUVECs (GFP-HUVECs, Angioproteomie, Boston, MA) and human lung fibroblasts (hLFs, Lonza) were cultured in endothelial growth medium 2 (EGM-2, Lonza) with GA1000 replaced with 1% penicillin and streptomycin (P/S, Thermo Fisher Scientific) and fibroblast growth medium 2 (FGM-2, Lonza), respectively. The cells were cultured in cell culture dishes in a humidified incubator at 37 °C and 5% CO_2_. The cells from passages four to six were used for the experiments.

To prepare the on-chip microvasculature, GFP-HUVECs and hLFs were prepared in EGM-2 at 1.6 × 10^7^ and 1 × 10^7^ cells/ml, respectively. Fibrinogen (Sigma), collagen type I (Corning), and aprotinin (Sigma) were mixed on ice to prepare a fibrin-collagen gel. The cells were then mixed with the fibrin-collagen gel solution in equal proportions such that the final concentrations of fibrinogen, collagen type I, and aprotinin in the gel–cell mixture were 2.5 mg/ml, 0.2 mg/ml, and 0.15 U/ml, respectively. Thrombin (Sigma, 0.5 U/ml) was added to the gel–cell solution prior to cell injection into the microfluidic device. Thirty microliters of GFP-HUVEC suspension (8 × 10^6^ cells/ml) was injected into channel three, and 20 *μ*l of hLF suspension (5 × 10^6^ cells/ml) was injected into channels one and five. The device was then placed in an incubator at 37 °C for 15 min to polymerize the gel. Subsequently, EGM-2 was added to the medium reservoirs and channels two and four, and the device was kept in an incubator at 37 °C and 5% CO_2_ in a humidified chamber. After two days, to allow the opening of the microvasculature, 20 *μ*l of GFP-HUVECs (5 × 10^6^ cells/ml) in EGM-2 was introduced into channel two and incubated at 37 °C and 5% CO_2_ for 30 min. The same procedure was repeated for channel four. The medium was changed every 2 days.

### C. Sodium selenite preparation and administration

Sodium selenite (Na_2_SeO_3_; Fujifilm, Wako, Japan) was prepared in phosphate-buffered saline (DPBS) according to the manufacturer's instructions. The required concentrations (3 nM, 15 nM, and 3 *μ*M) of SS were prepared in EGM-2 using appropriate dilutions of the initial stock solution. The SS in EGM-2 was introduced into channels two and four, and the medium reservoirs of 7-day-old microvascular networks were used for the experiment [Fig. 1(b)]. The day of SS introduction is denoted as day 0. The medium containing SS was changed every other day.

### D. Morphology, perfusability, cytotoxicity, and permeability measurements

To measure microvasculature morphology, GFP images acquired using confocal microscopy were post-processed to obtain the maximum intensity-projected images. The images were then Gaussian filtered and binarized. The vasculature area was then measured using ImageJ (NIH). The binary images were then skeletonized and analyzed to measure the number of branches, junctions, endpoints, and branch lengths. The number of branches per square millimeter was calculated by dividing the number of branches by the total area in square millimeters. The average vessel diameter was calculated by dividing the vasculature area by the product of the branch number and average branch length.

The perfusability of the microvasculature was tested by introducing a fluorescent dye, 10 *μ*M rhodamine B-conjugated dextran (Rhodamine dextran, Sigma; 70 kDa), in DPBS into channel two after removing EGM-2 from the reservoirs. The pressure difference between channels two and four allowed the perfusion of the dye through the microvascular networks, and the images were captured.

The cytotoxicity was measured by seeding GFP-HUVECs in at least three independent cell culture dishes for high (3 *μ*M) concentration after 24 h. The number of live and dead cells were measured using colorimetry using trypan blue staining (Thermo Fisher Scientific) (Fig. S1).

The permeability of the microvasculature was measured by injecting 10 *μ*l of 5 *μ*M rhodamine dextran in DPBS into channel two after removing EGM-2 from the medium reservoirs.^11^ Images were immediately taken using a confocal microscope every 6 s, and images at approximately 70 s were used for the measurement of permeability. This time point corresponds to saturated or stable intensity values inside the vessels. At least ten boxes of size 80 × 80 pixels were randomly chosen in each device such that each box included both the inside and outside regions of the vessels. Intensity values inside the vessel corresponding to GFP-positive areas and the values outside the vessel corresponding to GFP-negative areas were calculated separately using ImageJ. The ratio of intensity values outside to inside the vessels (out/in) was calculated and defined as dye leakage or permeability. Regions with no intensity values were considered outliers and were removed from further calculations.

### E. Reactive oxygen species (ROS) evaluation

ROS detection was performed using the ROS assay-highly sensitive DCFH DA kit (Dojindo, Japan) following the manufacturer's protocol. Briefly, after washing the preformed on-chip microvasculature twice with Hank's balanced salt solution (HBSS; Gibco), the working solution of the ROS assay was introduced into channels two and four (day 0) and incubated for 30 min. The working solution was then removed, the microvasculature was washed twice with HBSS, the medium containing SS (3 *μ*M) was added, and the chip was incubated for 1 h. Subsequently, the medium was removed, and the microvasculature was washed again with HBSS and imaged to observe fluorescence. Red fluorescent protein-labeled HUVECs were used in this experiment (Fig. S2).

### F. Quantitative PCR

Total RNA was extracted using the Qiagen RNeasy Micro Kit according to the manufacturer's protocols. cDNA was synthesized using the PrimeScript RT Master Mix (Takara Bio Inc., Japan) and a thermal cycler (Bio-Rad Lab, Inc., Tokyo, Japan). Quantitative RT-PCR was performed using TB Green Premix Ex Taq II (Takara Bio, Inc., Shiga, Japan), and β-actin (ACTB) was used as the housekeeping gene. The primers used are listed in Table I. For each condition, four independent replicates and four technical replicates were used. A fold change greater than or equal to 1.5 was considered as upregulation and less than or equal to 0.75 as downregulation.

**TABLE I.** List of primers for PCR.

Gene	Forward primer	Reverse primer
ACTB	CAATGTGGCCGAGGACTTTG	CATTCTCCTTAGAGAGAAGTGG
ERG	CAGCAGGATTGGCTGTCTCA	CATTCACCTGGCTAGGGTTACAT
CLDN5	CTCTGCTGGTTCGCCAACAT	CACAGACGGGTCGTAAAACTC
ANGPT1	TGCTGAACGGTCACACAGAG	CCCCCTCAAAGAAAGCGTTTG
ANGPT2	GAACCAGACGGCTGTGATGA	GGGAGTGTTCCAAGAGCTGA
CDH5	CTTCACCCAGACCAAGTACACA	AATGGTGAAAGCGTCCTGGT
FIGF	TCCCATCGGTCCACTAGGTT	TGGTACTCTTCCCCAGCTCA
TIE2	GCGAGATGGATAGGGCTTGA	GCACAGAAGCAGGCTGTAAC
VEGFA	ACGAAAGCGCAAGAAATCCC	CTCCAGGGCATTAGACAGCA
CD31	AAACCACTGCAGAGTACCAGG	GCCTCTTTCTTGTCCAGTGTC
TJP1	GTTTATTTGGGCTGTGGCGTG	TCCTCCATTGCTGTGCTCTTG
ECAD	CCTGGGACTCCACCTACAGA	TGGATTCCAGAAACGGAGGC

### G. Cell adhesion assay

GFP-HUVECs were plated on culture dishes with or without SS (3 *μ*M) and incubated at 37 °C and 5% CO_2_. After 4 to 7 h of plating, the cells were collected using a capillary-based vacuum-assisted cell and tissue acquisition system (UnipicK+, NeuroInDx, Inc., CA) with the following settings: force = 35.9 nN, acquisition time = 20 ms, and acquisition pressure = −12 kPa. Glass capillaries with diameters of 60 *μ*m were used. For three independent dishes under each condition, the percentage of detached (collected) cells (n >100) was calculated with respect to the total number of cells to which a vacuum pulse was applied.

### H. Image acquisition and statistical analysis

Images were captured using a confocal microscope (Olympus FV-3000, Tokyo, Japan). The images were binarized, skeletonized, and analyzed using ImageJ (NIH). All data are presented as the mean ± SD. Statistical tests were performed using the GraphPad Prism software (San Diego, CA, USA). Tukey's multiple comparison test was used for the significance test of percentage vasculature area, average diameter, and branches per square millimeter. Sidak's multiple comparison test was used for the significance test of percentage vasculature area and branches per square millimeter, and a two-tailed unpaired t-test was used for the significance testing of relative gene expression by RT-PCR, leakage ratio, and the percentage of detached cells. The following signifiers were used: ^*^p < 0.05, ^**^p < 0.01, ^***^p < 0.001, ^****^p < 0.0001, and ns = no significance. Schematic illustrations were prepared using BioRender, Adobe Illustrator, and AutoCAD Fusion 360 software.

## SUPPLEMENTARY MATERIAL

See the supplementary material for figures showing cytotoxicity of SS and the reactive oxygen species (ROS) evaluation on-chip.

## Data Availability

The data that support the findings of this study are available within the article and its supplementary material.
